# Natural Occurrence and Co-Contamination of Twelve Mycotoxins in Industry-Submitted Cool-Season Cereal Grains Grown under a Low Heat Unit Climate Condition

**DOI:** 10.3390/toxins11030160

**Published:** 2019-03-13

**Authors:** Haitao Shi, Warren Schwab, Peiqiang Yu

**Affiliations:** 1 Department of Animal and Poultry Science, College of Agriculture and Bioresources, University of Saskatchewan, 6D10 Agriculture Building, 51 Campus Drive, Saskatoon, SK S7N 5A8, Canada; shihaitao@swun.edu.cn; 2 Ministry of Education Key Laboratory of Conservation & Utilization of Qinghai-Tibetan Plateau Animal Genetic Resources, Southwest Minzu University, Chengdu 610041, China; 3 College of Life Science and Engineering, Foshan University, Guangdong 528000, China; 4 Prairie Diagnostic Services, University of Saskatchewan, Saskatoon, SK S7N 5B4, Canada; warren.schwab@pds.usask.ca

**Keywords:** mycotoxin contamination, cereals, feed and food co-occurrence, deoxynivalenol

## Abstract

This study aims to evaluate the prevalence of mycotoxins in industry-submitted cool-season barley and wheat grown under low heat unit climate conditions. Seventy-two barley samples and 83 wheat samples were submitted by producers and industry from May 2016 to May 2017. The concentrations of twelve common mycotoxins, including nivalenol (NIV), deoxynivalenol (DON), 3-acetyldeoxynivalenol (3-ADON), 15-acetyldeoxynivalenol (15-ADON), ochratoxin A (OTA), zearalenone (ZEN), α-zearalenol (α-ZAL), β-zearalenol (β-ZAL), diacetoxyscirpenol (DAS), T-2 toxin (T-2), HT-2 toxin (HT-2), and aflatoxin B1 (AFB1), were determined using the liquid chromatography/tandem mass spectrometry method. Mycotoxins were detected in 40 barley (56%) and 35 wheat (42%) samples submitted by producers and industry. DON showed the highest incidence in barley (44%) and wheat (33%). None of the barley samples contained detectable DAS and no wheat samples tested positive for α-ZAL, DAS, T-2, or AFB1. Co-occurrence of DON and other mycotoxins was frequently observed. Among the mycotoxin-positive samples, 70% of barley samples and 54% of wheat samples were co-contaminated with at least two mycotoxins. Four barley (6%) and five wheat (6%) samples contained levels of DON above 1000 μg/kg (regulatory level in diets for lactating dairy animals) and HT-2 content in five barley (7%) and four wheat (5%) samples exceeded 100 μg/kg (regulatory level in diets for cattle and poultry). Overall, contamination of these mycotoxins was more frequent and more severe in barley in comparison with wheat that was submitted by producers and industry. Comprehensive strategies, including the prevention of Fusarium toxins contamination, the routine monitoring of their prevalence, the detoxification of them in feed and food, as well as the inhibition of their absorption in the gastrointestinal tract, are highly required. A rapid detection method needs to be developed to screen mycotoxins in industry-submitted cool-season cereal grains.

## 1. Introduction

Mycotoxins are toxic secondary metabolites, mainly produced by fungi belonging to the genera of Fusarium, Penicillium, Aspergillus, and Alternaria [1,2]. Chemically, they exhibit diverse structures and have various biological effects on humans and animals, such as carcinogenicity, teratogenicity, mutagenicity, neurotoxicity, or immunotoxicity [2,3,4]. Mycotoxin contamination has been a worldwide problem for crop cultivation, animal production and human health for a long time. More than 300 mycotoxins have been reported, and only those that have been proven carcinogenic and/or toxic have received extensive scientific attention [5].

Fumonisins, trichothecenes, and zearalenone (ZEN) are common Fusarium toxins present in cereals. Toxins mainly produced by *Fusarium culmorum*, *F. graminearum*, *F. sporotrichioides*, and *F. poae*, trichothecenes are known to cause emesis, inflammation, and diarrhea [6,7]. Mycotoxins T-2, HT-2, DAS, DON, 3-ADON, 15-ADON, and NIV are commonly-detected trichothecenes in cereals. ZEN is produced by *F. culmorum* and *F. graminearum*. It is capable of binding estrogen receptors and inducing some estrogenic syndromes such as uterine enlargement, pseudopregnancy, swelling of mammary glands and vulva [3,6,7]. α-zearalenol (α-ZAL) and β-zearalenol (β-ZAL) are the most abundant derivatives of ZEN which are also exhibiting estrogenic activities [8,9]. Ochratoxin (OTA) is a mycotoxin produced by *Penicillium* and *Aspergillus* species and can be found in various food commodities [10]. As a potent nephrotoxin, OTA also possesses teratogenic, hepatotoxic, and immunosuppressive properties and is classified as a class 2B human carcinogen by the International Agency for Research on Cancer (IARC) [11,12]. Aflatoxins are mainly produced by *Aspergillus flavus* and *Aspergillus parasiticus* [13]. Among the known aflatoxins, aflatoxins B1 (AFB1) is considered as the most potent one which is highly teratogenic, carcinogenic, mutagenic, and immunosuppressive and has been classified as a Group 1 carcinogen by IARC [11].

Co-occurrence of mycotoxins in cereal and cereal-derived products has been observed in several studies. The main reason is that the same food or feed material can be colonized by various mycotoxigenic fungi that are capable of producing a variety of mycotoxins [14,15]. Wheat and barley are Canada’s most widely grown crops and represent the most contaminated crops [16,17]. While the study on the presence and co-occurrence of major mycotoxins in wheat and barley is still limited, knowledge regarding the occurrence and co-contamination of multiple mycotoxins in grain can further provide the basis for future toxicological studies on combined effects and the development of effective hazards control strategies.

Taking advantage of the reliable separation performance of liquid chromatography and the superior sensitivity of mass spectrometry, liquid chromatography-tandem mass spectrometry (LC-MS/MS) has been widely used as a powerful technique for the simultaneous determination of multiple mycotoxins in food and feed samples [18,19]. The objective of this study was to examine the recent presence of 12 mycotoxins and their natural co-occurrence patterns in industry-submitted cool-season barley and wheat samples collected by Prairie Diagnostic Services (PDS), University of Saskatchewan.

## 2. Results and Discussion

According to the results, 40 barley (56%) and 35 wheat (42%) samples were contaminated by mycotoxin. Thirty-two barley (44%) and 27 wheat (33%) samples were positive for DON; 3-ADON was detected in 17 barley (24%) and 12 wheat (14%) samples; 15 barley (21%) and nine wheat (11%) samples were contaminated with HT-2; seven barley (10%) and five wheat (6%) samples tested positive for 15-ADON; OTA was present in seven barley (10%) and four wheat (5%) samples; five barley (7%) samples were contaminated by NIV while only one wheat (1%) sample contained a detectable level of NIR; ZEN and β-ZAL were detected in two barley (3%) and one wheat (1%) samples; T-2 was found in six barley (8%) samples, α-ZAL and AFB_1_ were detected in one (1%) barley samples, while no wheat sample was contaminated with them. In this study, no barley or wheat sample was positive for DAS. A previous study reported that DON, ZEN, T-2, OTA, NIV, 3-ADON, and 15-ADON were also detected in barley and oats grown in Eastern Canada, while no sample contained detectable HT-2 [16].

In general, barley samples showed a higher incidence of these mycotoxins than wheat samples. This finding is consistent with previous research which reported that barley is more vulnerable to *Fusarium* species than wheat [16,20]. However, the average concentration (428.2 μg/kg) and median (62.8 μg/kg) of DON in DON-positive barley was lower than that in wheat samples (mean: 1026.9 μg/kg; median: 76.7 μg/kg), mainly because there were four highly contaminated wheat samples that contained high levels of DON (above 2 mg/kg).

In this study, DON was the most frequently detected mycotoxin while only five barley and one wheat samples contained detectable NIV at the levels ranging from 22.1 to 114.6 μg/kg. Previous studies reported that DON was the most frequently present *Fusarium* mycotoxin in wheat in some EU countries and NIV was the next most frequently detected one [21,22]. In another study, DON showed the highest incidence (72%) compared with other mycotoxins in barley samples collected during 1991 to 1998 from Eastern Canada where 34% (18/53) of the barley samples were contaminated with NIV [16]. According to a survey by Tittlemier et al. (2013), DON and NIV are the two most abundant toxins detected in Canadian grains and now represent the two major concerns for the safety of wheat and barley products [23].

DON was also reported as the mycotoxin with the highest incidence in feedstuffs of dairy cattle in the Netherlands [24]. Results from this study indicated that the prevalence of NIV could be affected by regions and continuous efforts should be taken to reduce DON contamination in cool-season barley and wheat in Western Canada. The contamination rate of OTA in wheat samples was lower than that reported in wheat from the previous study. Thirty-seven percent of samples collected from Canadian wheat shipments during 2011 and 2012 contained quantifiable OTA [17]. It should be noted that the LOQ of OTA (1 μg kg^−1^) was lower in their study and the median of OTA was only 2.10 μg kg^−1.^

The allowable limits for mycotoxins in feed and food are presently incomplete and contentious. It is well accepted that animal feed plays an important role in the food safety chain. According to regulatory guidance established by Canadian Food Inspection Agency (CFIA), four barley (6%) and five wheat (6%) samples contained levels of DON above 1000 μg/kg (regulatory level in diets for lactating dairy animals). A previous study reported that all the barley samples collected from Manitoba in 1993 and 1994 were positive for DON with mean content above 2.56 mg/kg and maximum level up to 15.79 mg/kg [25]. As explained by the authors, the heavy contamination of DON in barley samples was mainly because of the Southern Manitoba experienced exceptionally high midsummer rainfall in 1993 and 1994. The contamination rate and maximum level of DON were lower in this study. The content of HT-2 toxin in five barley (7%) and four wheat (5%) samples exceeded 100 μg/kg (regulatory level in diets for cattle and poultry). In addition, one barley sample contained AFB_1_ content above 20 μg/kg (regulatory level for animal feeding stuffs). None of the barley and wheat sales contained concentrations of OTA, ZEN, and T-2 that exceeded their guidance values. Based on the above findings, effective strategies, especially pre-harvest control strategies, are still required to prevent the growth of *Fusarium* fungi in barley and wheat since both DON and HT-2 are trichothecenes produced by them [1]. Caution should be used when incorporating higher proportions of these severely contaminated grains in animal diets.

Exploring the co-occurrence of mycotoxins in agricultural commodities can provide more comprehensive information on risk assessment. Among the mycotoxin-positive samples, 12 barley (30%) and 16 wheat (46%) samples contaminated with one mycotoxin; 13 barley (33%) and 13 wheat (37%) samples tested positive for two mycotoxins; seven barley (18%) and five wheat (14%) samples were found to be contaminated with three mycotoxins; five barley (13%) and one (3%) wheat samples contained four mycotoxins; three barley (8%) tested positive for five mycotoxins. Multiple mycotoxin contaminations were observed in 28 barley and 19 wheat samples.

**Figure 1:**
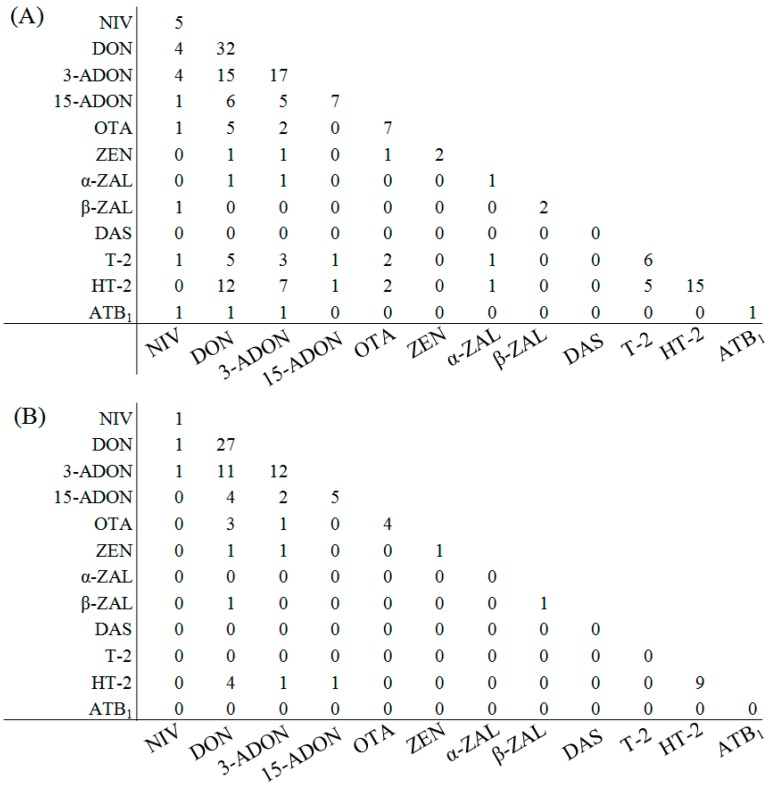
Co-occurrence of 12 mycotoxins in industry-submitted cool-season barley (**A**) and wheat (**B**) samples grown under cold climate condition (number of samples co-contaminated). NIV: Nivalenol; DON: Deoxynivalenol; 3-ADON: 3-Acetyldeoxynivalenol; 15-ADON: 15-Acetyldeoxynivalenol; OTA: Ochratoxin A; ZEN: Zearalenone; α-ZAL: α-Zearalenol; β-ZAL: β-Zearalenol; DAS: Diacetoxyscirpenol; T-2: T-2 toxin; HT-2: HT-2 toxin; AFB_1_: Aflatoxin B_1_.

Co-occurrence of mycotoxins was summarized in Figure 1. The co-occurrence of DON and its acetylated versions 3-ADON or 15-ADON was frequently observed in barley and wheat samples. Co-occurrence of DON and 3-ADON was observed in 15 barley and 11 wheat samples. A previous study reported that 3-ADON and 15-ADON are co-contaminants of DON and are only normally detected when DON is present at high levels [26]. In the present study, some samples with low DON levels (<100 μg/kg) were also tested positive for 3-ADON and/or 15-ADON. The result was consistent with the findings of a recent survey conducted in Central Canada by Burlakoti et al. (2017) [27]. The concentration of 15-ADON in six corn samples was greater than that of DON. In addition, 3-ADON and 15-ADON were also detected in both wheat and corn samples contaminated with low DON levels. These results indicated that the relationship between DON and its acetyl derivative might not be constant.

Co-contamination of DON and OTA was observed in five of seven OTA-positive barley samples and three of four OTA-positive wheat samples. It is reported that OTA does not normally present before harvest and is considered to be a problem of storage, while DON is mainly produced during the growing period [28]. Other researchers have also reported co-occurrence of DON and OTA in grains. Seven wheat and oat-based bran supplements samples (10.5%) from the Spanish market were co-contaminated with DON and OTA [29]. In another study, 9.1% of oats and other coarse grains, 7.1% of maize and maize products were co-contaminated with DON and OTA [30]. Tittlemier et al. (2013) studied the occurrence of *Fusarium* toxins in Canadian western amber durum wheat harvested in 2010 from Saskatchewan and Alberta [29]. Fifty-four durum wheat samples that contained *Fusarium*-damaged kernels were selected for mycotoxin and fungal analysis. Their results showed that all samples were contaminated with beauvericin and approximately 75% of the samples contained DON, beauvericin, and moniliformin. The high incidence of *Fusarium* damage and DON mainly resulted from the excessive precipitation on much of the Canadian Prairies in the 2010 growing year.

T-2 and HT-2 are closely related toxins since they are usually produced by the same fungi species [21]. In this study, five of the six T-2-positive (83%) barley samples were co-contaminated with HT-2. The natural co-occurrence of T-2 and HT-2 in wheat and barley samples from Europe has also been reported by other researchers [21,31]. Five of the six T-2 contaminated barley samples and 12 of the 15 HT-2-positive barley samples were co-contaminated with DON. Furthermore, both HT-2 and DON were observed in four of the six T-2-positive barley samples. Co-occurrence of DON, 3-ADON, 15-ADON, H-2, and HT-2 was observed in one barley sample. In agreement with this data, a previous study reported that T-2 and HT-2 often appear along with DON since all of them are derivatives of *Fusarium* species [32]. In the present study, one wheat sample was co-contaminated with DON, 3-ADON, 15-ADON, and HT-2. However, no T-2 was detected in wheat samples and only four of the nine HT-2-positive samples were co-contaminated with DON, indicated that the co-occurring patterns of these three mycotoxins could be affected by grain types.

## 3. Conclusions

The high prevalence and co-occurrence rate of *Fusarium* mycotoxins, especially DON and HT-2, in barley and wheat underlines the necessity of continuous monitoring of the major mycotoxins in different agricultural commodities and exploring the relative toxicity of individual mycotoxins and their combination in order to accurately evaluate feed and food quality and potential risks. Knowledge regarding the present and co-occurrence of multiple mycotoxins is still limited and current regulations on the safe levels of mycotoxins in feed and food do not address co-contamination issues and associated risks. Further research is expected to investigate the dynamics involving mycotoxin production, reduce their presence and content, and explore the possible additive or synergistic toxicity of them.

## 4. Material and Methods

### 4.1. Samples for Mycotoxin Analysis

In this study, 72 industry submitted barley samples and 83 industry submitted wheat samples (less than 1 kg original seeds per sample) grown under cold climate condition in Western Canada (provinces of Saskatchewan, Alberta, and Manitoba) were randomly collected from May 2016 to May 2017 by Prairie Diagnostic Services (PDS), University of Saskatchewan, Canada. Samples submitted to PDS usually reflect client (e.g., livestock producer, grain farmer, feed companies, etc.) needs and clinical diseases. Samples were kept in polyethylene bags and stored for less than one month at 4 °C before analysis.

### 4.2. Mycotoxin Analysis Methods

#### 4.2.1. Chemicals and Reagents

HPLC-grade methanol and acetonitrile were purchased from VWR scientific (Mississauga, Canada). Ammonium acetate was supplied by Fisher Scientific (Fair Lawn, NJ, USA). Two hundred and twenty-five MycoSep Trich cleanup cartridges from Romer Labs (Union, MO, USA) were used. Mycotoxin standards of NIV, DON, 3-ADON, 15-ADON, OTA, ZEN, α-ZAL, β-ZAL, HT-2, AFB_1_, DAS and T-2 were purchased from Romer Labs (Union, MO, USA). Stock solutions were prepared in acetonitrile to 100 μg/mL for DON, DAS, NIV, 3-ADON, 15-ADON, AFB_1_, ZEN, T-2 and HT-2. OTA, α-ZAL, β-ZAL were prepared to 10 μg/mL. From the individual stock standard solutions a standard curve mixture was prepared with the following concentrations: DON, DAS, NIV, 3-ADON, 15-ADON and AFB_1_ (200, 120 and 40 ng/mL); HT-2 and T-2 (2500, 1250 and 40 ng/mL); OTA (50, 30 and 10 ng/mL); α-ZAL and β-ZAL (250, 150 and 50 ng/mL); and ZEN (500, 300 and 100 ng/mL) in methanol and stored at −80 °C for 3 months.

#### 4.2.2. Preparation of Samples for Mycotoxin Analysis

Finely ground and homogenized subsamples of 20.0 g were extracted with 100 mL acetonitrile/H_2_O (84:16, *v*/*v*) and blended for 10 min on a stir plate and then filtered through a folded filter paper (Whatman #41, 15 cm; Whatman, Inc., Clifton, NJ, USA). Five milliliters of the filtrate were then passed through a MycoSep 225 cleanup cartridge. The eluate was evaporated and resuspended in 100 μL methanol/10 mM ammonium acetate (50:50, *v*/*v*). Before LC-MS/MS analysis the mycotoxins dissolved in mobile phase were filtered through a 0.45 μm Nylon filter (Whatman, Inc., Clifton, NJ, USA).

#### 4.2.3. HPLC-MS/MS Test Procedure

The HPLC-MS/MS analysis of mycotoxins was carried out by PDS (accredited by the Standards Council of Canada as a testing laboratory for specific tests). The HPLC-MS/MS procedure used for mycotoxin determination was based on that described by a previous study [18,19].

The Micromass Quattro Ultima^TM^ LC-MS/MS system (Waters, Milford, MA, USA) equipped with (ESI) source and an 1100 Series HPLC system (Agilent, Waldbronn, Germany) was used. Chromatographic separation was performed at 35 °C on a Hichrom C-18-column, 150 × 4.6 mm i.d., 5 μm particle size, equipped with a C18 security guard cartridge (Rheodyne, Cotati, CA, USA). The injection volume was 20 μL. The mobile phase A (water/methanol, 65:35 (*v*/*v*)) and mobile phase B (methanol/water, 90:10 (*v*/*v*)) at flow rate of 0.4 mL/min with a gradient elution program. The gradient elution started at 100% mobile phase A with a linear decrease to 0% in 7 min and held for 0.1 min. During the next 10 min, mobile phase A was increased back to 100%. Initial column conditions were reached at 17 min.

The mass spectrometer was operated in the positive electrospray ionization (ESI+) mode for DAS, T-2, HT-2, and AFB_1_. Negative electrospray ionization (ESI-) mode was used for DON, NIV, 3-ADON, 15-ADON, ZEN, OTA, α-ZEL and β-ZEL. Capillary voltage was 3 kV, the cone was 35 and nitrogen was used as spray gas. Source and desolvation temperatures were set at 150 °C and 300 °C, respectively. In order to optimize the MS parameters, tuning solutions for each compound were infused continuously to the electrospray interface which recorded the incoming compound. Tuning solutions (100 ng/mL) were freshly prepared in mobile phase A/mobile phase B (50:50, *v*/*v*). The cone voltage for every compound was optimized. To enhance the sensitivity for all the mycotoxins, production scans (MS/MS spectra of the selected precursor ion) were carried out in 30 s using several collision energies. These mass spectra allowed to select the two most abundant product ions and optimize the corresponding collision energies. Mycotoxins were analyzed using selected reaction monitoring (SRM) channels. The LC retention time and the intensity ratio of the two MRM transitions agreed with the related values of an authentic standard within 0.1 min and 30% rel., respectively.

After separation and detection by LC-MS/MS, the calibration curves were generated in MassLynx 4.1 software using the equation *y* = *ax* + *b*, where *y* is analyte peak area abundance and *x* is the analyte concentration. The method was validated by determining the matrix effect, limit of detection (LOD), limit of quantitation (LOQ), the linearity of the calibration, recoveries, and repeatability (% RSD). LOD and LOQ were calculated by 3 times and 6 times, respectively, the standard error of the intercept divided by the slope of the calibration curve. For each component, the calculated LOD and LOQ were verified by the signal-to-noise (S/N) ratio which should be more than 3 and 10 according to the IUPAC settings. The apparent recovery was estimated by quantifying the mycotoxins using the calibration plot. The observed value was for each mycotoxin divided by the spiked level.

The LOD for DAS, NIV, DON, 3-ADON, 15-ADON, AFB_1_, T-2 and HT-2 was 13 μg/kg; OTA was 3 μg/kg; ZEN was 30 μg/kg; α-ZAL and β-ZAL was 17 μg/kg. The LOQ for DAS, NIV, DON, 3-ADON, 15-ADON, AFB_1_, T-2 and HT-2 was 40 μg/kg; OTA was 10 μg/kg; ZEN was 100 μg/kg; α-ZAL, and β-ZAL was 50 μg/kg.

### 4.3. Statistical Analysis

Only samples with mycotoxin concentrations above LOD were considered as positive samples and included in further co-occurrence analysis. PROC MEANS procedure in SAS (version 9.4, SAS Institute, Cary, NC, USA) was performed to calculate descriptive statistics of the mycotoxins. The mean, minimum, maximum, median and standard deviation (SD) of the 12 mycotoxins content in barley and wheat are presented in Table 1 and Table 2, respectively.

## Figures and Tables

**Table 1:** Descriptive statistics for 12 mycotoxins content in industry-submitted cool-season barley samples grown under cold climate condition.

Mycotoxin ^1^	No. of Samples	Positive Samples n (%)	Concentration (μg/kg) ^2^				
			Min	Max	Mean	Median	SD ^3^
NIV	72	5 (7%)	22.1	114.6	57.5	48.8	39.6
DON	72	32 (44%)	13.8	6880.7	428.2	62.8	1243.0
3-ADON	72	17 (24%)	15.8	164.6	46.4	25.0	42.1
15-ADON	72	7 (10%)	15.2	69.0	36.6	34.1	19.4
OTA	72	7 (10%)	6.9	127.2	64.8	53.5	37.7
ZEN	72	2 (3%)	957.7	962.8	960.3	960.3	3.6
α-ZAL	72	1 (1%)	-	-	26.5	-	-
β-ZAL	72	2 (3%)	46.5	92.7	69.6	69.6	32.7
DAS	72	0	-	-	-	-	-
T-2	72	6 (8%)	19.8	56.8	33.3	30.8	13.1
HT-2	72	15 (21%)	19.8	467.6	107.6	63.3	121.5
AFB_1_	72	1 (1%)	-	-	26.5	-	-
Total	72	40 (56%)	22.1	7090.8	482.8	132.5	1156.4

^1^ Abbreviation: NIV, nivalenol; DON, deoxynivalenol; 3-ADON, 3-acetyldeoxynivalenol; 15-ADON, 15-acetyldeoxynivalenol; OTA, ochratoxin A; ZEN, zearalenone; α-ZAL, α-zearalenol; β-ZAL, β-zearalenol; DAS, diacetoxyscirpenol; T-2, T-2 toxin; HT-2, HT-2 toxin; AFB_1_, aflatoxin B_1_. ^2^ Values of positive samples. ^3^ SD = Standard deviation.

**Table 2:** Descriptive statistics for 12 mycotoxins content in industry-submitted wheat samples grown under cold climate condition.

Mycotoxin ^1^	No. of Samples	Positive Samples n (%)	Concentration (μg/kg) ^2^				
			Min	Max	Mean	Median	SD ^3^
NIV	83	1 (1%)			22.7		
DON	83	27 (33%)	14.2	13,823.9	1026.9	76.7	2718.6
3-ADON	83	12 (14%)	15.6	187.0	75.4	47.2	60.5
15-ADON	83	5 (6%)	19.4	148.6	69.0	34.0	56.7
OTA	83	4 (5%)	22.1	43.5	32.6	32.5	9.1
ZEN	83	1 (1%)	-	-	126.0	-	-
α-ZAL	83	0	-	-	-	-	-
β-ZAL	83	1 (1%)	-	-	45.1	-	-
DAS	83	0	-	-	-	-	-
T-2	83	0	-	-	-	-	-
HT-2	83	9 (11%)	17.3	873.2	166.4	49.8	272.9
AFB_1_	83	0	-	-	-	-	-
Total	83	35 (42%)	17.3	14,017.5	880.0	76.7	2445.2

^1^ Abbreviation: NIV, nivalenol; DON, deoxynivalenol; 3-ADON, 3-acetyldeoxynivalenol; 15-ADON, 15-acetyldeoxynivalenol; OTA, ochratoxin A; ZEN, zearalenone; α-ZAL, α-zearalenol; β-ZAL, β-zearalenol; DAS, diacetoxyscirpenol; T-2, T-2 toxin; HT-2, HT-2 toxin; AFB_1_, aflatoxin B_1_. ^2^ Values of positive samples. ^3^ SD = Standard deviation.
